# Supplementary material for: Multi-level phenotypic models of cardiovascular disease and obstructive sleep apnea comorbidities: A longitudinal Wisconsin sleep cohort study
Source: PLoS One. 2025 Jul 15;20(7):e0327977. doi: 10.1371/journal.pone.0327977 (PMC12262892; doi:10.1371/journal.pone.0327977)
Supplement: S1 Table — (DOCX) [file pone.0327977.s001.docx]

**S1 Table. Description of variables.**

| **Variable** | **Description** |
| --- | --- |
| **Clinical Data** |  |
| total_cholesterol | Measure of total cholesterol level in blood, obtained from fasting blood draw on the morning after the overnight PSG |
| ldl | LDL Cholesterol: Measure of LDL level in blood, obtained from fasting blood draw on the morning after the overnight PSG |
| creatinine | Measure of creatinine level in blood, obtained from fasting blood draw on the morning after the overnight PSG |
| triglycerides | Measure of triglyceride level in blood, obtained from fasting blood draw on the morning after the overnight PSG |
| sbp_mean | Mean of seated systolic measures: Mean of two standard seated blood pressure measurements on the night of the PSG. If repeat measurements were taken, the mean of the closest two measurements. |
| uric_acid | Measure of uric acid level in blood, obtained from fasting blood draw on the morning after the overnight PSG |
| **Anthropometry** |  |
| bmi | Body mass index (BMI) |
| waisthip | Waist to hip ratio |
| neckgirthm | Mean of neck girth measures |
| hipgirthm | Mean of hip girth measures |
| **General Health** |  |
| eval_health | In general, would you say your health is (check one):  1: Excellent 2: Very good 3: Good 4: Fair 5: Poor |
| zung12_scored | Zung Depression Scale Item: I find it easy to do the things I used to do.  4: None or a little of the time 3: Some of the time 2: A good part of the time 1: Most or all of the time |
| zung_index | Standardized Zung Depression Scale Total Score: Scaled Zung Depression Scale score used with clinical cutpoints |
| **Medical History** |  |
| diabetes_med | Diabetes Medication/Insulin: Self-reported use Self-reported drugs at PSG visit, open text then grouped according to effect |
| arthritis_ynd | Self-reported diagnosis by a physician. The next section asks about specific medical problems. Please indicate if you have been told by a doctor that you have or have had any of these conditions: Arthritis |
| **Sleep Monitoring** | |
| mean_desat_perc | Average Level of Oxygen Desaturation of Apnea and Hypopnea Event: (Apneas with no oxygen desaturation threshold used and with or without arousal and hypopneas with discernible flow reduction and with >= 4% oxygen desaturation and with or without arousal) from type I polysomnography. |
| avgo2sattst | Average oxygen saturation in total sleep duration from type I polysomnography. Conventionally, total sleep duration would be called total sleep time (TST) (i.e. total time spent in stage 1 or greater). |
| ahi | Apnea-Hypopnea Index: (Apneas with no oxygen desaturation threshold used and with or without arousal and hypopneas with discernible flow reduction and with >= 4% oxygen desaturation and with or without arousal) / hours of sleep from type I polysomnography |
| nremahi | Apnea-Hypopnea Index (NREM): (Apneas with no oxygen desaturation threshold used and with or without arousal and hypopneas with discernible flow reduction and with >= 3% oxygen desaturation and with or without arousal)/ hours of NREM sleep from type I polysomnography. Calculated - Summary metric of sleep disordered breathing events during NREM sleep |
| **Sleep Questionnaires** | |
| apnea | Sleep Apnea: Self-reported diagnosis by a physician. Have you ever been told by a doctor that you have sleep apnea? |
| **Sleep Treatment** | |
| apnea_treatment | Sleep Apnea: Self-reported treatment recommendation 1. If yes, what treatment was recommended? (When told "Y" need treatment for sleep apnea) |
