# Supplementary material for: Multi-level phenotypic models of cardiovascular disease and obstructive sleep apnea comorbidities: A longitudinal Wisconsin sleep cohort study
Source: PLoS One. 2025 Jul 15;20(7):e0327977. doi: 10.1371/journal.pone.0327977 (PMC12262892; doi:10.1371/journal.pone.0327977)
Supplement: S2 Table — (DOCX) [file pone.0327977.s002.docx]

**S2 Table.** **Classification based on Total Cholesterol, Triglyceride, AHI and BMI.**

|  | **Level** | **Category** |
| --- | --- | --- |
| Total Cholesterol | Less than 200 mg/dl | Desirable level |
|  | 200 to 239 mg/dl | Borderline high for heart disease |
|  | 240 mg/dl and above | High blood cholesterol. A person with this level has more than twice the risk of heart disease as someone whose cholesterol is below 200 mg/dl. |
| Triglyceride | Less than 150 mg/dl | Normal |
|  | 150-199 mg/dl | Borderline high |
|  | 200-499 mg/dl | High |
|  | 500 mg/dl and above | Very high |
| AHI | <5 | Normal |
|  | 5-15 | Mild obstructive |
|  | 15-30 | Moderate |
|  | >30 | Severe |
| BMI | Below 18.5 | Underweight |
|  | 18.5-24.9 | Healthy Weight |
|  | 25.0-29.9 | Overweight |
|  | 30.0 and above | Obesity |
