# Supplementary material for: Multi-level phenotypic models of cardiovascular disease and obstructive sleep apnea comorbidities: A longitudinal Wisconsin sleep cohort study
Source: PLoS One. 2025 Jul 15;20(7):e0327977. doi: 10.1371/journal.pone.0327977 (PMC12262892; doi:10.1371/journal.pone.0327977)
Supplement: S3 Table — (DOCX) [file pone.0327977.s003.docx]

**S3 Table.** **Comparative analysis of variables for healthy patients from Visit 2 who tend to move to Cluster 1 or Cluster 2 in Visit 3 within Group 1.**

|  | **G1V2HV3C1** | | **G1V2HV3C2** | | | |
| --- | --- | --- | --- | --- | --- | --- |
|  | **Visit2** | **Visit 3**  **(Cluster1)** | **Visit2** | | **Visit 3**  **(Cluster2)** | |
|  | **Subjects = 4** | | **Subjects =9** | | | |
| Cholesterol medication | No, N=4 100% | Yes, N=4  100% | No, N=5 55.56% | Yes,N=4  44.44% | No, N=2  22.22% | Yes, N=7  77.78% |
| MACE1 | 0 | 4(100) | 0 | 0 | 1(50) | 5(71.43) |
| MACE1 treatment | 0 | 3(75) | 0 | 0 | 1(50) | 1(14.28) |
| MACE3 | 0 | 0 | 0 | 0 | 1(50) | 2(28.57) |
| MACE3 treatment | 0 | 0 | 0 | 0 | 0 | 0 |
| apnea | 0 | 0 | 0 | 1(25) | 1(50) | 3(42.86) |
| apnea treatment | 0 | 0 | 0 | 1(25) | 1(50) | 3(42.86) |
| total cholesterol | 236.75  (20.42) | 130.50  (14.20) | 224.00  (53.16) | 189.00  (21.37) | 221.00  (43.84) | 169.00  (22.11) |
| ldl | 160.25  (31.33) | 71.75  (13.62) | 130.20  (58.83) | 104.50  (16.74) | 107.50  (2.12) | 89.29  (13.49) |
| triglycerides | 111.75  (62.71) | 61.75  (22.34) | 258.60  (115.33) | 138.75  (4.50) | 358.00  (247.49) | 172.43  (41.18) |
| nremahi | 3.95  (4.47) | 4.83  (4.01) | 11.36  (10.28) | 11.18  (7.96) | 19.05  (17.04) | 11.19  (10.34) |
| ahi | 4.05  (4.10) | 5.28  (2.53) | 16.00  (13.10) | 13.73  (4.05) | 26.15  (21.71) | 12.59  (9.23) |
| hipgirthm | 100.58  (6.13) | 97.58  (6.36) | 121.96  (14.69) | 121.95  (20.68) | 135.50  (21.92) | 116.37  (12.90) |
| diabetes_med | 0 | 0 | 2(40) | 1(25) | 1(50) | 3(42.86) |
| arthritis_ynd | 0 | 0 | 4(80) | 1(25) | 2(100) | 5(71.43) |
| bmi | 27.73  (3.16) | 26.48  (2.26) | 36.48  (6.06) | 39.38  (11.44) | 42.00  (8.63) | 34.84  (7.37) |
| age | 57.50  (4.80) | 61.50  (4.43) | 59.80  (12.32) | 57.75  (6.34) | 53.50  (0.71) | 66.29  (9.27) |
| creatine | 1.15  (0.19) | 1.01  (0.22) | 0.98  (0.30) | 1.03  (0.17) | 0.86  (0.20) | 1.06  (0.32) |
| waitsthip | 0.91  (0.09) | 0.95  (0.08) | 0.92  (0.09) | 0.96  (0.12) | 0.92  (0.06) | 0.98  (0.07) |
| Zung index | 37.81  (5.44) | 38.13  (4.27) | 43.25  (9.30) | 41.88  (7.81) | 56.88(7.95) | 41.96(5.04) |
