# Supplementary material for: Multi-level phenotypic models of cardiovascular disease and obstructive sleep apnea comorbidities: A longitudinal Wisconsin sleep cohort study
Source: PLoS One. 2025 Jul 15;20(7):e0327977. doi: 10.1371/journal.pone.0327977 (PMC12262892; doi:10.1371/journal.pone.0327977)
Supplement: S4 Table — (DOCX) [file pone.0327977.s004.docx]

**S4 Table. Comparative analysis of variables for healthy patients from Visit 1 who tend to move to Cluster 1 or Cluster 2 in Visit 2 within Group 3.**

|  | **G3V1HV2C1** | | **G3V1HV2C2** | | | |
| --- | --- | --- | --- | --- | --- | --- |
|  | **Visit1** | **Visit 2**  **(Cluster 1)** | **Visit1** | | **Visit 2**  **(Cluster 2)** | |
|  | **Subjects = 4** | | **Subjects = 5** | | | |
| Cholesterol  medication | No, N=4  (100%) | Yes, N=4  (100%) | No, N=4  (80%) | Yes, N=1  (20%) | No, N=1  (20%) | Yes, N=4  (80%) |
| MACE1 | 0 | 3 (75) | 0 | 0 | 1 | 3(75) |
| MACE1 treatment | 0 | 4(100) | 0 | 0 | 0 | 4(100) |
| MACE3 | 1(25) | 0 | 2 | 0 | 1 | 1(25) |
| MACE3 treatment | 0 | 0 | 0 | 0 | 0 | 1(25) |
| apnea | 1(25) | 1(25) | 1(25) | 1 | 1 | 2(50) |
| apnea treatment | 1(25) | 1(25) | 1(25) | 1 | 1 | 2(50) |
| total cholesterol | 209.33  (21.73) | 133.00  (11.17) | 203.25  (47.68) | 216.00 | 169.00 | 162.00  (19.85) |
| ldl | 130.00(28.35) | 59.25(7.27) | 116.50(49.20) | 144.00 | 105.00 | 88.25(29.43) |
| triglycerides | 176.00  (95.09) | 89.00  (17.26) | 230.25  (143.22) | 154.00 | 148.00 | 201.00  (137.64) |
| nremahi | 21.53  (25.49) | 22.58  (21.86) | 36.28(49.14) | 2.20 | 5.20 | 29.60(40.38) |
| ahi | 23.67  (24.85) | 24.60  (22.40) | 39.73(48.74) | 2.80 | 10.70 | 31.55(38.67) |
| hipgirthm | 98.43  (9.40) | 99.83(6.34) | 116.63(11.16) | 102.30 | 133.00 | 110.45  (12.04) |
| diabetes_med | 0 | 1(25) | 0 | 1 | 0 | 1(25) |
| arthritis_ynd | 1(25) | 1(25) | 2(50) | 0 | 1 | 2(50) |
| bmi | 26.93(3.00) | 26.40(4.34) | 35.63(6.01) | 30.60 | 41.20 | 34.28(6.96) |
| age | 60.67(8.62) | 66.00(7.87) | 59.50(9.18) | 65.00 | 72.00 | 62.25(7.04) |
| creatine | 1.27(0.06) | 1.10(0.08) | 1.03(0.21) | 1.10 | 1.40 | 0.96(0.16) |
| waitsthip | 1.00(0.06) | 0.98(0.10) | 0.95(0.09) | 1.02 | 0.89 | 0.97(0.12) |
| Zung index | 42.50(6.50) | 42.50(8.10) | 46.25(18.74) | 45.00 | 33.75 | 41.25(10.46) |
