# Supplementary material for: Multi-level phenotypic models of cardiovascular disease and obstructive sleep apnea comorbidities: A longitudinal Wisconsin sleep cohort study
Source: PLoS One. 2025 Jul 15;20(7):e0327977. doi: 10.1371/journal.pone.0327977 (PMC12262892; doi:10.1371/journal.pone.0327977)
Supplement: S5 Table — (DOCX) [file pone.0327977.s005.docx]

**S5 Table.** **Comparative analysis of variables for patients from Visit 1 who tend to move to Cluster 1 or Cluster 2 in Visit 2 within Group 7.2 and Group 7.3.**

|  | **G7V1C2V2C1** | | | **G7V1C2V2C2** | |
| --- | --- | --- | --- | --- | --- |
|  | **Visit1**  **(Cluster 2)** | | **Visit 2**  **(Cluster 1)** | **Visit1**  **(Cluster 2)** | **Visit 2**  **(Cluster 2)** |
|  | **Subjects = 7** | | | **Subjects =6** | |
| Cholesterol  medication | No, N=1  14.29% | Yes, N=6  85.71% | Yes, N=7  100% | Yes, N=6  100% | Yes, N=6  100% |
| MACE1 | 1 | 6(100) | 7(100) | 5(83.33) | 5(83.33) |
| MACE1 treatment | 1 | 4(66.67) | 7(100) | 5(83.33) | 5(83.33) |
| MACE3 | 0 | 2(33.33) | 2(28.57) | 2(33.33) | 3(50) |
| MACE3 treatment | 0 | 0 | 0 | 0 | 0 |
| apnea | 1 | 0 | 2(28.57) | 0 | 1(16.67) |
| apnea  treatment | 1 | 0 | 2(28.57) | 0 | 1(16.67) |
| total cholesterol | 157.00 | 170.33(30.69) | 146.43(22.86) | 173.67(24.38) | 162.50(20.90) |
| ldl | 76.00 | 84.67(18.92) | 72.00(23.01) | 86.83(25.24) | 82.00(18.92) |
| triglycerides | 239.00 | 179.67(33.69) | 106.14(25.16) | 232.17(51.13) | 189.00(52.49) |
| nremahi | 22.10 | 14.80(21.57) | 14.13(10.19) | 21.18(30.19) | 14.55(18.90) |
| ahi | 23.20 | 18.48(21.74) | 17.07(11.35) | 23.18(32.00) | 17.30(21.76) |
| hipgirthm | 112.80 | 105.93(9.99) | 104.54(6.08) | 115.05(5.79) | 111.13(10.88) |
| diabetes_med | 0 | 1(16.67) | 2(28.57) | 3(50) | 4(66.67) |
| arthritis_ynd | 1 | 2(33.33) | 4(57.14) | 3(50) | 2(33.33) |
| bmi | 33.40 | 29.63(4.39) | 30.00(3.73) | 36.42(5.77) | 35.32(7.30) |
| age | 70.00 | 59.50(7.31) | 65.14(7.67) | 61.00(6.54) | 65.00(6.57) |
| creatine | 1.30 | 1.05(0.20) | 1.04(0.19) | 0.90(0.15) | 0.98(0.18) |
| waitsthip | 1.01 | 0.97(0.07) | 0.99(0.07) | 1.00(0.12) | 1.01(0.11) |
| Zung index | 38.75 | 43.54(6.63) | 40.54(8.00) | 43.54(8.85) | 42.92(5.16) |
