# Supplementary material for: Multi-level phenotypic models of cardiovascular disease and obstructive sleep apnea comorbidities: A longitudinal Wisconsin sleep cohort study
Source: PLoS One. 2025 Jul 15;20(7):e0327977. doi: 10.1371/journal.pone.0327977 (PMC12262892; doi:10.1371/journal.pone.0327977)
Supplement: S6 Table — (DOCX) [file pone.0327977.s006.docx]

**S6 Table. Comparative analysis of variables for patients from Visit 2 who tend to move to Cluster 1 or Cluster 2 in Visit 3 within Group 7.2 and Group 7.3.**

|  | **G7V2C1V3C1** | | **G7V2C1V3C2** | |
| --- | --- | --- | --- | --- |
|  | **Visit 2**  **(Cluster 1)** | **Visit 3**  **(Cluster 1)** | **Visit 2**  **(Cluster 1)** | **Visit 3 (Cluster2)** |
|  | Subjects = 4 | | Subjects =3 | |
| Cholesterol medication | Yes, N=4  100% | Yes, N=4  100% | Yes, N=3  100% | Yes, N=3  100% |
| MACE1 | 4(100) | 4(100) | 3(100) | 3(100) |
| MACE1 treatment | 4(100) | 4(100) | 3(100) | 3(100) |
| MACE3 | 2(50) | 2(50) | 0 | 0 |
| MACE3 treatment | 0 | 0 | 0 | 0 |
| apnea | 2(50) | 3(75) | 0 | 0 |
| apnea treatment | 2(50) | 2(50) | 0 | 0 |
| total cholesterol | 139.75(20.09) | 123.50(14.55) | 155.33(27.47) | 158.33(15.89) |
| ldl | 65.25(9.22) | 56.25(9.32) | 81.00(35.34) | 88.00(16.37) |
| triglycerides | 104.50(35.29) | 102.00(13.98) | 108.33(4.16) | 171.67(68.54) |
| nremahi | 11.45(9.64) | 18.80(29.21) | 17.70(11.77) | 19.53(21.73) |
| ahi | 12.93(8.56) | 20.43(29.06) | 22.60(14.00) | 25.03(22.19) |
| hipgirthm | 107.20(1.80) | 107.20(1.59) | 101.00(8.54) | 101.17(10.25) |
| diabetes_med | 2(50) | 2(50) | 0 | 0 |
| arthritis_ynd | 2(50) | 3(75) | 2(66.67) | 2(66.67) |
| bmi | 30.80(2.18) | 31.13(1.67) | 28.93(5.62) | 29.97(6.43) |
| age | 67.75(5.68) | 71.50(5.32) | 61.67(9.81) | 66.67(9.81) |
| creatine | 1.08(0.25) | 1.11(0.28) | 1.00(0.10) | 1.03(0.33) |
| waitsthip | 1.00(0.04) | 1.05(0.06) | 0.96(0.11) | 1.02(0.09) |
| Zung index | 37.81(8.92) | 41.25(10.75) | 44.17(6.17) | 41.25(9.44) |
