# Supplementary material for: Multi-level phenotypic models of cardiovascular disease and obstructive sleep apnea comorbidities: A longitudinal Wisconsin sleep cohort study
Source: PLoS One. 2025 Jul 15;20(7):e0327977. doi: 10.1371/journal.pone.0327977 (PMC12262892; doi:10.1371/journal.pone.0327977)
Supplement: Attachment — Submitted filename: Review 2.pdf [file pone.0327977.s007.pdf]

Review

Manuscript Title:

Multi-level Phenotypic Models of Cardiovascular Disease and Obstructive Sleep Apnea Comorbidities: A Longitudinal Wisconsin Sleep Cohort Study

Summary of Manuscript:

Objective:
This study investigates the dynamic relationship between cardiovascular diseases (CVD) and obstructive sleep apnea (OSA) using a novel multi-level phenotypic modeling approach. By leveraging longitudinal data from the Wisconsin Sleep Cohort, the study employs logistic mixed-effects modeling (LGMM), t-distributed stochastic neighbor embedding (t-SNE), and Gaussian Mixture Models (GMM) to identify distinct phenotypic clusters and predict disease progression.

Key Findings:
The authors demonstrate the feasibility of stratifying OSA-CVD phenotypes into high- and low-risk groups for major adverse cardiovascular events (MACEs). The LGMM achieved a diagnostic accuracy of 95.56%, and the clustering analysis revealed critical clinical predictors, including nocturnal hypoxia, cholesterol levels, and diabetes. These findings hold significant potential for improving personalized interventions in OSA-CVD comorbidities.

Major Comments:

1. Study Design and Methods:

Strengths:
- The integration of longitudinal data and advanced modeling techniques is a major strength.
- The use of LGMM, combined with dimensionality reduction (t-SNE) and clustering (GMM), is innovative and aligns well with the study’s objective.

Recommendations:
- Attrition Bias: High attrition rates (e.g., 748 participants at Visit 2 vs. 121 at Visit 4) may introduce bias. The manuscript should explicitly discuss strategies employed to handle missing data (e.g., imputation techniques or inverse probability weighting). Including sensitivity analyses would enhance confidence in the findings.
- Clustering Validation: The robustness of GMM clustering is not validated using external metrics such as silhouette scores or Davies-Bouldin index. Adding these metrics would strengthen the credibility of the identified clusters.

- Generalizability: The cohort is largely middle-aged and homogenous, limiting the applicability of findings to broader populations. Discuss the need for external validation in diverse ethnic, socioeconomic, and geographic groups.

2. Statistical Analysis:

Strengths:
- The statistical framework effectively captures the longitudinal progression of OSA-CVD comorbidities and accounts for inter-individual variability.
- The inclusion of tree-based feature importance analysis adds transparency to the model's predictive approach.

Recommendations:
- SMOTE (Synthetic Minority Over-sampling Technique), while helpful in addressing class imbalance, may introduce bias. Discuss its potential influence on model outcomes and provide validation to ensure generalizability.

3. Results Interpretation:

Strengths:
- The results are well-interpreted and contextualized within existing literature, particularly emphasizing the role of nocturnal hypoxia and lipid dysregulation in CVD progression.

Recommendations:
- Expand on the mechanistic pathways connecting OSA and CVD, particularly the role of intermittent hypoxia, oxidative stress, and autonomic dysfunction.
- Provide more explicit discussion on how phenotypes influence treatment strategies (e.g., CPAP therapy or lipid-lowering medications). This would bridge the findings to practical applications.

4. Clinical Relevance:

Strengths:
- The phenotypic clusters have clear clinical utility, particularly in stratifying patients for personalized interventions and early risk detection.

Recommendations:
- Propose actionable recommendations for clinical practice, such as thresholds for monitoring nocturnal hypoxia or specific lipid levels based on phenotypic risk stratification.
- Highlight potential integration into clinical workflows, such as embedding the model into

electronic health records or decision-support tools to guide patient management.

Minor Comments:

1. Introduction:
The introduction is comprehensive but could further highlight gaps in existing predictive models and emphasize how this study addresses those gaps.

2. Mechanistic Insights:
While the study identifies important predictors, it lacks a deeper exploration of their biological roles. Including a brief discussion on the interplay between OSA severity, vascular changes, and metabolic dysregulation would enhance the narrative.

3. Limitations Section:
The limitations section should discuss potential confounders, such as lifestyle factors (e.g., smoking, alcohol use, and caffeine intake), which were not integrated into the analysis. Additionally, self-reported data for certain variables should be acknowledged as a limitation.

Recommendations:

Revisions Required:
To strengthen the manuscript, the following revisions are recommended:
1. Include clustering validation metrics (e.g., silhouette scores).
2. Address missing data and attrition bias with detailed sensitivity analyses.
3. Expand on the mechanistic and clinical implications of phenotypes.

Potential for Publication:
With these revisions, the manuscript is highly suitable for publication in PLOS ONE. Its innovative methodology and clinically relevant findings contribute significantly to the understanding of OSA-CVD interactions.

Overall Assessment:

This manuscript is scientifically rigorous, methodologically sound, and clinically important. With minor revisions, it will make a valuable contribution to the literature on OSA-CVD comorbidities and advance personalized medicine in this field. However, the above recommendations should not preclude publication.
