# Supplementary material for: Multi-level phenotypic models of cardiovascular disease and obstructive sleep apnea comorbidities: A longitudinal Wisconsin sleep cohort study
Source: PLoS One. 2025 Jul 15;20(7):e0327977. doi: 10.1371/journal.pone.0327977 (PMC12262892; doi:10.1371/journal.pone.0327977)
Supplement: Attachment — Submitted filename: Reviewer_response_PONE-D-24-44385_finalJan28.docx [file pone.0327977.s009.docx]

Dear Prof. Amir Hossein Behnoush and Reviewers,

Thank you for your constructive feedback and valuable suggestions on our manuscript entitled

“***Multi-level Phenotypic Models of Cardiovascular Disease and Obstructive Sleep Apnea Comorbidities: A Longitudinal Wisconsin Sleep Cohort Study***” (Manuscript Ref: PONE-D-24-44385). We are grateful for the opportunity to enhance our work based on the reviewers’ and your insights. In response to the feedback, we have undertaken a comprehensive revision of our manuscript:

- **Introduction Expanded:** Added context on existing models and research gaps.
- **Results Clarified:** Adjusted interpretations to align with established medical knowledge.
- **Discussion Enhanced:** Included clinical utility of findings and integrated actionable recommendations for clinical practice.
- **Reviewer Comments Addressed:** Abbreviations defined, typographical errors corrected, incorporated relevant meta-analyses, and expanded discussion on mechanistic pathways of OSA and CVDs.
- **Methodological Refinements:** Discussed data handling strategies and the influence of SMOTE on model outcomes.

Please find attached a detailed point-by-point response to each reviewer's comments, outlining how we have addressed their concerns and suggestions. The updates in the revised manuscript are highlighted in **blue** text in this response to the reviewer document for easy reference. We believe that these revisions have significantly improved the manuscript. We hope that the changes meet your expectations and that the manuscript is now suitable for publication in *PLOS ONE*.

We look forward to the possibility of our work contributing to the journal and the broader community.

With best regards,

Phat

**Phat Kim Huynh**, Ph.D.

**Assistant Professor**

Department of Industrial and Systems Engineering

North Carolina A&T State University

Phone: (469) 816-2177

Email: [pkhuynh@ncat.edu](mailto:pkhuynh@ncat.edu)

Website: [www.PassioLab.com](http://www.passiolab.com/)

# Response to Reviewer 1

***Reviewer’s comment:***

**#1A - Define abbreviations in their first use and make sure that abbreviated forms are used after the definition.**

***Response****:*

We thank the reviewer for your helpful comment. In response, we have thoroughly reviewed the manuscript and ensured that all abbreviations are defined at their first mention in the text. Following the definition, the abbreviated forms are consistently used throughout the manuscript. Additionally, to enhance clarity and provide a quick reference for readers, we have included a **Table of Nomenclature** in the manuscript (located after the Abstract in the revised manuscript).

| **Abbreviation** | **Definition** |
| --- | --- |
| AHI | Apnea-Hypopnea Index |
| BIC | Bayesian Information Criterion |
| BMI | Body Mass Index |
| CI | Confidence Interval |
| CVD | Cardiovascular Disease |
| GMM | Gaussian Mixture Model |
| HDL | High-density Lipoprotein |
| KLD | Kullback-Leibler Divergence |
| KNN | K-Nearest Neighbor |
| LDL | Low-density Lipoprotein |
| LGMM | Logistic Gaussian Mixture Model |
| LR | Logistic Regression |
| MACE | Major Adverse Cardiovascular Event |
| NREM-AHI | Non-rapid Eye Movement Apnea-Hypopnea Index |
| OSA | Obstructive Sleep Apnea |
| SD | Standard Deviation |
| SE | Standard Error |
| SMOTE | Synthetic Minority Over-sampling Technique |
| t-SNE | t-distributed Stochastic Neighbor Embedding |
| WSCS | Wisconsin Sleep Cohort Study |

***Reviewer’s comment:***

**#1B - I found some typos and grammatical errors. A native review is warranted.**

***Response****:*

We thank the reviewer for highlighting this issue. We carefully reviewed the manuscript for typos and grammatical errors and have made the necessary corrections.

***Reviewer’s comment:***

**#1C - Use previous meta-analyses on the association between apnea and other lipid and insulin resistance markers including LAP, VAI, AIP, and TyG.**

***Response****:*

We thank the reviewer for this insightful suggestion. In response, we have incorporated relevant findings from previous meta-analyses addressing the association between OSA and lipid and insulin resistance markers such as the Lipid Accumulation Product (LAP), Visceral Adiposity Index (VAI), Atherogenic Index of Plasma (AIP), and the TyG index. These findings have been added to the **Discussion** section to contextualize our results within the broader literature and to highlight the role of these metabolic markers in the progression of OSA and cardiovascular disease. Specifically, we referenced studies that demonstrate the predictive value of these markers for metabolic syndrome and cardiovascular risk, further emphasizing their relevance to our study’s findings. The following text has been added to **Section IV: Discussion**.

Given that the association between OSA and CVD extends to several comorbidities, including obesity, dyslipidemia, and diabetes, incorporating data on metabolic syndrome biomarkers, such as Lipid Accumulation Product (LAP), Visceral Adiposity Index (VAI), Atherogenic Index of Plasma (AIP), and the insulin resistance marker TyG index, could enhance our understanding of OSA-CVD progression and its predictors [85, 86].

***Reviewer’s comment:***

**#1D - Add the clinical utility of your findings to the discussion.**

***Response****:*

We thank the reviewer for highlighting the importance of elaborating on the clinical utility of our findings. To address this comment comprehensively, we have made substantial additions to the manuscript, incorporating detailed discussions of clinical implications as addressed in other specific reviewer comments. Below is a summary of the key changes as per the comments.

1. **Interpretation of Cholesterol and LDL Levels:** We clarified the biological and pharmacological context of counterintuitive findings regarding reduced cholesterol and LDL levels, linking them to confounding factors such as medication use. This discussion is now included in **Section III - Part B** and is further supported by detailed analyses in **Section III – Part C** of **Tables A3-A6** in the Appendix. These insights highlight how cholesterol management influences patient trajectories and outcomes.

**Section III – B**

Total cholesterol *(“total_cholesterol”)* showed a significant negative association with CVD outcomes (estimate: -0.0081, p-value: 0.0031). This counterintuitive finding likely reflects the use of cholesterol-lowering medications, such as statins, prescribed in response to high cholesterol levels. Statins reduce the risk of cardiovascular events and have been recommended as a primary prevention of CVD [50].

HDL cholesterol showed a positive association with CVD outcomes (estimate: 0.0325, p-value: 0.0013). While HDL is traditionally considered cardioprotective due to its role in reverse cholesterol transport, recent studies suggest that dysfunctional HDL, characterized by impaired antioxidative and anti-inflammatory properties, may increase CVD risk in both healthy and clinical populations [51, 52].

LDL cholesterol was negatively associated with CVD outcomes (estimate: -0.0113, p-value: <0.001). While elevated cholesterol and LDL levels are widely recognized as risk factors for CVD, the reduced levels observed in certain clusters may reflect a more complex relationship between lipid profiles, disease progression, and treatment interventions. In patients with CVD, lower cholesterol levels might result from long-term statin therapy or other cholesterol-lowering treatments aimed at reducing cardiovascular risk [53].

Intriguingly, the model for Group 1 showed that LDL cholesterol levels had a small negative association with CVD outcomes. This minimal effect size is likely attributable to confounding factors, such as the use of cholesterol-lowering therapies, rather than indicating a direct biological relationship. Additionally, the use of diabetes medications may confound these relationships. Such medications not only improve lipid profiles but also introduce anti-inflammatory and insulin-sensitizing effects, which could further complicate the interpretation of the observed associations between cholesterol levels and CVD outcomes.

**Section III - C**

**Table A3** shows the analysis of patients in the pre-CVD stage at Visit 2. At this stage, patients tend to progress into one of the two identified CVD states: Cluster 1 (Cardiovascular Stability Cluster) or Cluster 2 (Cardiovascular Risk Cluster). We defined **G1V2HV3C1** as patients at Visit 2 likely to progress towards Cluster 1 at Visit 3, and **G1V2HV3C2** as patients at Visit 2 who tend to move towards Cluster 2 at Visit 3. Overall, patients in **G1V2HV3C1** showed a better response to cholesterol treatment, maintaining a healthier profile with fewer comorbidities and lower AHI levels, supporting their classification as CVD-healthy in Cluster 1. In contrast, **G1V2HV3C2** patients experienced more health complications, with higher comorbidity rates, limited improvement in lipid profiles, and worsening triglyceride levels, indicating a decline in cardiovascular health outcomes in Cluster 2.

In group **G1V2HV3C1**, all patients were in a healthy status at Visit 2 and received no cholesterol treatment despite elevated levels of cholesterol (236.75 mg/dl), LDL (160.25 mg/dl), and triglycerides (111.75 mg/dl). By Visit 3, all patients have started cholesterol medication, resulting in significant reductions in lipid levels to 130.50 mg/dl, 71.75 mg/dl, and 61.75 mg/dl, respectively. AHI levels remained low across the pre-CVD stage and the CVD stage in Cluster 1. Between Visit 2 and Visit 3, the mean AHI increased from 4.05 to 5.28, while the NREM-AHI rose from 3.95 to 4.83. At Visit 3, the patients transitioned to the CVD stage in Cluster 1, with 75% receiving CVD treatment.

In group **G1V2HV3C2**, at Visit 2, 55.56% of patients without cholesterol treatment exhibited higher levels of cholesterol (224.50 ± 53.16 mg/dl), LDL (130.20 ± 58.83 mg/dl), and triglycerides (258.60 ± 115.33 mg/dl) compared to the those receiving treatment (cholesterol: 189.00 ± 21.37 mg/dl, LDL: 140.50 ± 16.74 mg/dl, triglycerides: 138.75 ± 4.50 mg/dl). Both groups shared similar constitutions, including comorbidities such as diabetes, arthritis, and OSA. They also displayed mid-obstructive to moderate AHI levels and elevated BMI levels, classifying many individuals as obese. These factors suggest that both groups face an increased risk of developing CVD over time, with a tendency towards poorer health outcomes. By Visit 3, all patients transitioned to Cluster 2, with 77.78% receiving cholesterol treatment. Among the treatment group, although there was a slight reduction in cholesterol levels (189.00 to 169.00 mg/dl) and LDL levels (104.05 to 89.29 mg/dl), triglyceride levels significantly increased from 138.75 to 172.43 mg/dl. For the 22.22% not receiving cholesterol treatment, cholesterol and LDL levels showed little change, but triglycerides rose significantly from 258.60 to 358.00 mg/dl, underscoring treatment’s impact on lipid levels. These trends, also observed in Table A4 for the two sub-groups: **G3V1HV2C1** (patients with no CVD at Visit 1 but had CVD in Cluster 1 at Visit 2) and **G3V1HV2C2** (patients with no CVD at Visit 1 but had CVD in Cluster 2 at Visit 2), suggest that treatment improves lipid metabolism, potentially benefiting cardiovascular health. Therefore, the reduced cholesterol and LDL levels can be considered confounding factors.

**Tables A5 and A6** present the analysis of patients in the long-term CVD group (Group 7), comparing the following categories: patients who transitioned from Cluster 2 to Cluster 1 (**G7V1C2V2C1)**, patients who remained in Cluster 2 across both visits (**G7V1C2V2C2)**, patients who transitioned from Cluster 1 to Cluster 2 (**G7V2C1V3C2)**, and patients who remained in Cluster 1 across both visits (**G7V2C1V3C1)**. **Table A5** focuses on the sub-groups **G7V1C2V2C1** and **G7V1C2V2C2**, while **Table A6** focuses on sub-groups as **G7V2C1V3C2** and **G7V2C1V3C1**. In **Table A5**, both sub-groups received cholesterol treatment, resulting in similar lipid levels except for triglycerides, which showed a significant difference. For individuals transitioning from Cluster 2 to Cluster 1, the triglyceride level was 179.67 md/dl, while for those remaining in Cluster 2, it was significantly higher at 232.17 mg/dl. A similar pattern was observed for NREM-AHI and AHI levels: transitioning patients had lower NREM-AHI (14.80) and AHI (18.48) compared to those remaining in Cluster 2 (NREM-AHI: 21.18, AHI: 23.18). This suggests that transitioning from Cluster 2 to Cluster 1 is associated with improvements in lipid and AHI, reflecting a potential shift towards better overall health. Conversely, individuals remaining in Cluster 2 exhibit persistently higher triglycerides and worse respiratory parameters, highlighting continued health risks.

In **Table A6**, all patients received cholesterol treatment. Patients who remained in Cluster 1 had lower AHI, NREM-AHI, and BMI values at Visit 2 compared to Visit 3. This indicates that failure to maintain these metrics within a healthy range increases the risk of transitioning to Cluster 2, a poorer health state. Individuals transitioning to Cluster 2 exhibited higher lipid and AHI levels compared to those remaining in Cluster 1. This suggests that maintaining these indicators at lower levels, as observed in patients transitioning from Cluster 2 to Cluster 1 in Table A5, is associated with better overall health. Conversely, an increase in these indicators among individuals remaining in Cluster 2 or moving towards it could be a sign of deteriorating health risk, particularly related to respiratory disorders and lipid metabolism. This highlights the crucial role of medical interventions in controlling these indicators to prevent health decline.

2. **Practical Implications of Clustering Analysis:** We updated **Section IV: Discussion** to outline how the identified phenotypic clusters inform treatment strategies. Specifically:
   - **Cluster 1 (Cardiovascular Stability):** Recommendations focus on maintaining current preventive measures, such as routine monitoring and conservative management.
   - **Cluster 2 (Cardiovascular Risk):** Recommendations emphasize more aggressive interventions, including lipid-lowering therapies, glucose control, and CPAP therapy for OSA management. These additions bridge the clustering findings to actionable clinical strategies.

The following paragraphs have been revised in the Discussion in the revised manuscript:

Our clustering analysis identifies two distinct patient groups with direct implications for clinical decision-making:

- ***Cardiovascular Stability Cluster* (Cluster 1):** This cluster includes patients with more favorable health profiles, characterized by lower LDL cholesterol, fasting glucose, and fewer episodes of nocturnal hypoxia, indicating stable disease progression and effective management of CVD and OSA. These findings suggest the importance of maintaining current treatment regimens and focusing on preventive care, such as lifestyle modifications and routine monitoring. Patients in Cluster 1, characterized by slower CVD progression, may benefit from more conservative treatment approaches, with careful monitoring of CPAP adherence and standard cardiovascular risk management [81].
- ***Cardiovascular Risk Cluster* (Cluster 2):** This cluster includes patients with higher values of critical CVD biomarkers and more severe OSA symptoms, reflecting rapid disease progression and a need for intensive management strategies. For these patients, more aggressive lipid-lowering therapies, stricter glucose control, and potentially earlier initiation of lipid-lowering medications [82] as well as targeted interventions for sleep apnea, such as CPAP therapy, may be necessary.

These clustering insights support precision medicine by stratifying patients based on risk, enhancing decision-support tools to flag high-risk phenotypes, suggest personalized interventions, and prioritize resource allocation. For instance, patients identified in high-risk clusters could automatically trigger alerts or recommendations for further diagnostic tests or follow-up care.

3. **Actionable Recommendations and Thresholds:** We proposed specific thresholds for lipid and respiratory metrics based on phenotypic risk stratification, providing clinicians with practical guidelines to monitor and intervene effectively. This is detailed in **Section IV: Discussion**, supported by analysis in **Tables A5 and A6**.

Monitoring nocturnal hypoxia, defined as oxygen saturation below 90% of sleep time, can also aid in identifying high-risk individuals, as prolonged nocturnal desaturation is associated with increased risks of CVD [83]. OSA screening is recommended for patients with poorly controlled hypertension, heart failure, recurrent atrial fibrillation, or a history of stroke, with CPAP therapy prioritized for severe OSA cases. Integrating risk stratification and treatment strategies into routine cardiovascular practice not only supports personalized care but also addresses the under-recognition of OSA and its critical role in CVD outcomes.

Furthermore, the temporal progression patterns identified in our study could inform the timing and intensity of interventions, allowing clinicians to proactively adjust treatment plans based on a patient's phenotypic classification. Building on these insights, the analysis of Tables A5 and A6 provides the following actionable recommendations for patients in Group 7 (long-term CVD):

- **Cluster 1 recommendations**: Patients in Cluster 1 should focus on maintaining their current metrics within a healthy range, including lipid levels, AHI, and NREM-AHI. Regular check-ups and proactive management of these indicators are important to prevent a potential transition to Cluster 2.
- **Cluster 2 recommendations:** Patients in Cluster 2 should undergo regular monitoring and targeted medical interventions to control lipid levels and improve respiratory health. To facilitate the transition to Cluster 1, patients should focus on comprehensive lifestyle changes, including adopting a heart-healthy diet, increasing physical activity, and managing stress, as well as adopting specific treatments as needed for cholesterol management, weight loss, or respiratory metrics. We recommend that patients aim for the following target values: total cholesterol between 123.50 mg/dL and 155.33 mg/dL, with a target of 139.42 mg/dL; LDL between 56.25 mg/dL and 81 mg/dL, with a target of 68.63 mg/dL; triglycerides between 102.00 mg/dL and 108.33 mg/dL, with a target of 105.17 mg/dL; and AHI between 12.93 and 22.60, with a target of 17.77.

Recent research indicates that extremely low or high LDL-C levels might be linked to higher mortality rates. LDL levels below 50 mg/dL or above 130 mg/dL have been linked to a higher risk of mortality in patients with coronary artery disease [84], highlighting the relationship between extreme LDL levels and all-cause death rates. Additionally, severe OSA (AHI ≥30) has been associated with a higher risk of both all-cause and cardiovascular mortality [3]. However, it is important to note that the baseline characteristics are not fully aligned across the sub-groups. This heterogeneity limits our ability to define specific thresholds for clusters or to provide definitive recommendations regarding remaining in or transitioning to a preferable healthy cluster. Consequently, while we can offer general suggestions on managing key indicators such as lipid levels and respiratory health based on our findings, further studies with more consistent baseline data are needed to establish clearer thresholds and provide precise recommendations for achieving optimal cluster outcomes.

4. **Integration into Clinical Workflows:** In **Section IV: Discussion**, we elaborated on how our modeling framework could be embedded into clinical workflows, such as electronic health records and decision-support tools. These insights enable dynamic tracking of patient trajectories, flagging high-risk phenotypes, and supporting personalized interventions.

Our proposed research, which combines the LGMM for patient trajectory analysis and clustering using t-SNE and GMM, holds significant potential for integration into clinical workflows. Specifically, the Logistic Mixed-Effects Model could be embedded into electronic health records (EHR) utilizing 20 selected features to provide predictions of CVD outcomes, enabling clinicians to dynamically track patient trajectories and make timely interventions. t-SNE and GMM-based clustering results could further classify patients into phenotypic groups directly within EHR systems, offering a visual and data-driven understanding of patient profiles.

5. **Mechanistic Pathways Connecting OSA and CVD:** We added a detailed explanation of the mechanistic links, including the roles of intermittent hypoxia, oxidative stress, and autonomic dysfunction, to the **Discussion section**. This strengthens the biological basis of our findings and highlights their relevance to clinical decision-making.

Mechanistically, the interplay between OSA severity, vascular dysfunction, and metabolic dysregulation provides insight into the observed CVD progression patterns. Recurrent intermittent hypoxia induces oxidative stress and systemic inflammation, leading to endothelial dysfunction and arterial stiffness [79], while sleep fragmentation disrupts glucose homeostasis through increased insulin resistance [80]. These synergistic pathways - vascular inflammation, sympathetic overactivity, and metabolic dysfunction - may explain the aggressive disease trajectory in Cluster 2. In particular, patients in Cluster 2 likely experienced heightened sympathetic activation and severe nocturnal hypoxemia, intensifying the inflammatory cascade and metabolic disruption, which resulted in their observed rapid onset and deterioration of cardiovascular conditions compared to Cluster 1.

Response to Reviewer 2

***Reviewer’s comment:***

**#2A - However, certain aspects raise questions. For instance, the interpretation of reduced cholesterol levels and LDL as potentially protective factors in CVD seems counterintuitive and demands further biological explanation. This point could benefit from a clearer discussion, as it appears to contradict established medical knowledge.**

***Response****:*

We thank the reviewer for the insightful comments. We have revised the interpretation of total cholesterol and LDL levels as protective factors and clarified the observed negative association of CVD outcomes and lipid indicators. Specifically, we have elaborated on how these findings likely reflect confounding factors, such as cholesterol-lowering and diabetes treatments, supported by plausible biological mechanisms consistent with established medical knowledge. We have carefully addressed this comment in the revised manuscript in **Section III – B** as follows:

Total cholesterol (*“total_cholesterol”)* showed a significant negative association with CVD outcomes (estimate: -0.0081, p-value: 0.0031). This counterintuitive finding likely reflects the use of cholesterol-lowering medications, such as statins, prescribed in response to high cholesterol levels. Statins reduce the risk of cardiovascular events and have been recommended as a primary prevention of CVD [50]

HDL cholesterol showed a positive association with CVD outcomes (estimate: 0.0325, p-value: 0.0013). While HDL is traditionally considered cardioprotective due to its role in reverse cholesterol transport, recent studies suggest that dysfunctional HDL, characterized by impaired antioxidative and anti-inflammatory properties, may increase CVD risk in both healthy and clinical populations [51, 52].

LDL cholesterol was negatively associated with CVD outcomes (estimate: -0.0113, p-value: <0.001). While elevated cholesterol and LDL levels are widely recognized as risk factors for CVD, the reduced levels observed in certain clusters may reflect a more complex relationship between lipid profiles, disease progression, and treatment interventions. In patients with CVD, lower cholesterol levels might result from long-term statin therapy or other cholesterol-lowering treatments aimed at reducing cardiovascular risk [53].

Intriguingly, the model for Group 1 showed that LDL cholesterol levels had a small negative association with CVD outcomes. This minimal effect size is likely attributable to confounding factors, such as the use of cholesterol-lowering therapies, rather than indicating a direct biological relationship. Additionally, the use of diabetes medications may confound these relationships. Such medications not only improve lipid profiles but also introduce anti-inflammatory and insulin-sensitizing effects, which could further complicate the interpretation of the observed associations between cholesterol levels and CVD outcomes.

We have also included a detailed analysis of subjects’ transition based on Figure 5 in the manuscript’s **Appendix**, focusing on cholesterol medication monitoring in the groups developing CVD and with long-term CVD, to address the reviewer’s feedback more comprehensively. This content has been mentioned to answer reviewer 1’s comment, we included the content here for the completeness of the response.

**Section III - C**

We have also conducted a detailed analysis of subjects’ transition based on **Figure 5**, focusing on cholesterol medication monitoring in the groups developing CVD and with long-term CVD. **Table A3** shows the analysis of patients in the pre-CVD stage at Visit 2. At this stage, patients tend to progress into one of the two identified CVD states: Cluster 1 (Cardiovascular Stability Cluster) or Cluster 2 (Cardiovascular Risk Cluster). We defined **G1V2HV3C1** as patients at Visit 2 likely to progress towards Cluster 1 at Visit 3, and **G1V2HV3C2** as patients at Visit 2 who tend to move towards Cluster 2 at Visit 3. Overall, patients in **G1V2HV3C1** showed a better response to cholesterol treatment, maintaining a healthier profile with fewer comorbidities and lower AHI levels, supporting their classification as CVD-healthy in Cluster 1. In contrast, **G1V2HV3C2** patients experienced more health complications, with higher comorbidity rates, limited improvement in lipid profiles, and worsening triglyceride levels, indicating a decline in cardiovascular health outcomes in Cluster 2.

In group **G1V2HV3C1**, all patients were in a healthy status at Visit 2 and received no cholesterol treatment despite elevated levels of cholesterol (236.75 mg/dl), LDL (160.25 mg/dl), and triglycerides (111.75 mg/dl). By Visit 3, all patients have started cholesterol medication, resulting in significant reductions in lipid levels to 130.50 mg/dl, 71.75 mg/dl, and 61.75 mg/dl, respectively. AHI levels remained low across the pre-CVD stage and the CVD stage in Cluster 1. Between Visit 2 and Visit 3, the mean AHI increased from 4.05 to 5.28, while the NREM-AHI rose from 3.95 to 4.83. At Visit 3, the patients transitioned to the CVD stage in Cluster 1, with 75% receiving CVD treatment.

In group **G1V2HV3C2**, at Visit 2, 55.56% of patients without cholesterol treatment exhibited higher levels of cholesterol (224.50 ± 53.16 mg/dl), LDL (130.20 ± 58.83 mg/dl), and triglycerides (258.60 ± 115.33 mg/dl) compared to the those receiving treatment (cholesterol: 189.00 ± 21.37 mg/dl, LDL: 140.50 ± 16.74 mg/dl, triglycerides: 138.75 ± 4.50 mg/dl). Both groups shared similar constitutions, including comorbidities such as diabetes, arthritis, and OSA. They also displayed mid-obstructive to moderate AHI levels and elevated BMI levels, classifying many individuals as obese. These factors suggest that both groups face an increased risk of developing CVD over time, with a tendency towards poorer health outcomes. By Visit 3, all patients transitioned to Cluster 2, with 77.78% receiving cholesterol treatment. Among the treatment group, although there was a slight reduction in cholesterol levels (189.00 to 169.00 mg/dl) and LDL levels (104.05 to 89.29 mg/dl), triglyceride levels significantly increased from 138.75 to 172.43 mg/dl. For the 22.22% not receiving cholesterol treatment, cholesterol and LDL levels showed little change, but triglycerides rose significantly from 258.60 to 358.00 mg/dl, underscoring treatment’s impact on lipid levels. These trends, also observed in Table A4 for the two sub-groups: **G3V1HV2C1** (patients with no CVD at Visit 1 but had CVD in Cluster 1 at Visit 2) and **G3V1HV2C2** (patients with no CVD at Visit 1 but had CVD in Cluster 2 at Visit 2), suggest that treatment improves lipid metabolism, potentially benefiting cardiovascular health. Therefore, the reduced cholesterol and LDL levels can be considered confounding factors.

**Tables A5 and A6** present the analysis of patients in the long-term CVD group (Group 7), comparing the following categories: patients who transitioned from Cluster 2 to Cluster 1 (**G7V1C2V2C1)**, patients who remained in Cluster 2 across both visits (**G7V1C2V2C2)**, patients who transitioned from Cluster 1 to Cluster 2 (**G7V2C1V3C2)**, and patients who remained in Cluster 1 across both visits (**G7V2C1V3C1)**. **Table A5** focuses on the sub-groups **G7V1C2V2C1** and **G7V1C2V2C2**, while **Table A6** focuses on sub-groups as **G7V2C1V3C2** and **G7V2C1V3C1**. In **Table A5**, both sub-groups received cholesterol treatment, resulting in similar lipid levels except for triglycerides, which showed a significant difference. For individuals transitioning from Cluster 2 to Cluster 1, the triglyceride level was 179.67 md/dl, while for those remaining in Cluster 2, it was significantly higher at 232.17 mg/dl. A similar pattern was observed for NREM-AHI and AHI levels: transitioning patients had lower NREM-AHI (14.80) and AHI (18.48) compared to those remaining in Cluster 2 (NREM-AHI: 21.18, AHI: 23.18). This suggests that transitioning from Cluster 2 to Cluster 1 is associated with improvements in lipid and AHI, reflecting a potential shift towards better overall health. Conversely, individuals remaining in Cluster 2 exhibit persistently higher triglycerides and worse respiratory parameters, highlighting continued health risks.

In **Table A6**, all patients received cholesterol treatment. Patients who remained in Cluster 1 had lower AHI, NREM-AHI, and BMI values at Visit 2 compared to Visit 3. This indicates that failure to maintain these metrics within a healthy range increases the risk of transitioning to Cluster 2, a poorer health state. Individuals transitioning to Cluster 2 exhibited higher lipid and AHI levels compared to those remaining in Cluster 1. This suggests that maintaining these indicators at lower levels, as observed in patients transitioning from Cluster 2 to Cluster 1 in Table A5, is associated with better overall health. Conversely, an increase in these indicators among individuals remaining in Cluster 2 or moving towards it could be a sign of deteriorating health risk, particularly related to respiratory disorders and lipid metabolism. This highlights the crucial role of medical interventions in controlling these indicators to prevent health decline.

**#2B - Similarly, while the clustering analysis is technically robust, its practical implications for clinical decision-making remain somewhat abstract and require stronger contextualization.**

***Response****:*

We appreciate your comment related to the practical implications of the clustering analysis. While the clustering analysis is technically robust, we acknowledge that its immediate clinical implications are limited, as the findings are specific to the dataset used. However, to highlight the practical implications of the clustering, we have added the following paragraphs to provide a stronger contextualization of our work to **Section IV: Discussion:**

Our clustering analysis identifies two distinct patient groups with direct implications for clinical decision-making:

- **Cardiovascular stability cluster (Cluster 1):** This cluster includes patients with more favorable health profiles, characterized by lower LDL cholesterol, fasting glucose, and fewer episodes of nocturnal hypoxia, indicating stable disease progression and effective management of CVD and OSA. These findings suggest the importance of maintaining current treatment regimens and focusing on preventive care, such as lifestyle modifications and routine monitoring. Patients in Cluster 1, characterized by slower CVD progression, may benefit from more conservative treatment approaches, with careful monitoring of CPAP adherence and standard cardiovascular risk management [81].
- **Cardiovascular risk cluster (Cluster 2):** This cluster includes patients with higher values of critical CVD biomarkers and more severe OSA symptoms, reflecting rapid disease progression and a need for intensive management strategies. For these patients, more aggressive lipid-lowering therapies, stricter glucose control, and potentially earlier initiation of lipid-lowering medications [82] as well as targeted interventions for sleep apnea, such as CPAP therapy, may be necessary.

***Reviewer’s comment:***

**#2C - The study’s attention to detail and depth of analysis are commendable, but the dense and highly technical language might limit accessibility for a broader scientific audience. The findings, although significant, would be more impactful if the conclusions were structured more clearly, highlighting practical applications and clinical recommendations.**

***Response****:*

We thank the reviewer for your constructive feedback regarding the presentation of our findings. In response to your comments, we have undertaken several revisions to enhance the accessibility and clarity of our manuscript:

- We have carefully reviewed the manuscript to simplify the language, reducing the use of technical jargon where possible without compromising the scientific integrity of the study.
- We have restructured the conclusions to emphasize the key findings and their implications in a more concise and impactful manner. The revised conclusion now explicitly outlines how the findings can be applied in clinical settings, suggesting specific strategies for the management of patients with obstructive sleep apnea and cardiovascular disease comorbidities based on the phenotypic clusters identified.

We have updated the **Conclusion section** in the revised manuscript, as shown below.

Our study elucidates the complex interplay between OSA and CVDs through a longitudinal analysis utilizing the WSCS. Our findings not only clarify the mechanisms underlying these comorbid conditions but also enhance predictive accuracies which could significantly impact clinical practices. Firstly, our research demonstrates the potent influence of specific biomarkers such as total cholesterol, LDL, and indicators of diabetes on the progression of CVDs in the presence of OSA. These factors serve as critical predictors within our logistic mixed-effects models, offering clinicians robust tools to identify at-risk patients early in the disease progression timeline. Moreover, the phenotypic clusters identified through our analysis provide a nuanced understanding of patient risk profiles. These clusters categorize patients into distinct groups with differing risks and progression patterns of disease, enabling personalized treatment approaches. Patients in high-risk clusters may benefit from aggressive management strategies including advanced CPAP therapy and targeted lipid-lowering treatments, whereas those in more stable clusters might be managed with routine monitoring and conservative care strategies.

The practical applications of our findings are substantial and have significant implications for clinical practice. Specifically, our research emphasizes the need for tailored therapeutic strategies that specifically consider the complex dynamics between OSA and cardiovascular diseases CVDs. By identifying and delineating the various phenotypic profiles associated with these comorbidities, clinicians can develop more personalized treatment plans that directly address the unique risk factors presented by individual patients. Our findings suggest that patients with severe OSA and concurrent high cardiovascular risk may benefit from an integrated treatment approach that includes aggressive management of sleep apnea with CPAP therapy, alongside proactive cardiovascular risk mitigation strategies such as statin therapy and lifestyle modifications. Conversely, patients identified with less severe forms of OSA without significant cardiovascular risk markers might be managed with less intensive interventions, focusing on lifestyle changes and regular monitoring. Moreover, our study's approach aids in the efficient allocation of healthcare resources by enabling healthcare providers to prioritize interventions for patients who are most at risk. This targeted intervention strategy not only improves the cost-effectiveness of healthcare delivery but also maximizes patient outcomes by preventing the progression of disease with precisely timed therapeutic interventions.

# Response to Reviewer 3

**Major Comments:**

***Reviewer’s comment:***

**#3A - Attrition Bias: High attrition rates (e.g., 748 participants at Visit 2 vs. 121 at Visit 4) may introduce bias. The manuscript should explicitly discuss strategies employed to handle missing data (e.g., imputation techniques or inverse probability weighting). Including sensitivity analyses would enhance confidence in the findings.**

***Response****:*

We thank the reviewer for your comment. We acknowledge the potential for attrition bias given the significant drop in participants from Visit 2 (748 participants) to Visit 4 (121 participants). To address this, we took careful steps to handle missing data and mitigate any potential biases. Specifically, we excluded Visits 4 and 5 and focused on patients who had data from Visits 1, 2, and 3, resulting in a cohort of 360 patients with complete data for these three visits. This ensured that there were no missing patient records for the selected visits.

In addition, we appreciate your valuable comment regarding the handling of missing data in our research. In response, we have implemented and compared different techniques to effectively address this issue. The steps are outlined below:

1. **Comparison of Inverse Probability Weighting and Imputation Techniques**
2. **Imputation Techniques**: We have applied both Multiple Imputation by Chained Equations (MICE) and k-Nearest Neighbors (KNN) imputation to handle missing feature values.
3. **Sensitivity analyses**: We have conducted sensitivity analyses for the KNN imputation method, including results for different parameter values (K=1, K=3, K=5), to identify the optimal configuration.
1. **Comparison of Inverse Probability Weighting and Imputation Techniques**

We have added the rationale for choosing KNN to handle missing data in **Section II: Methodology**.

To address the issue of missing data, we considered two approaches. Inverse Probability Weighting (IPW) adjusts observation weights based on missingness probabilities, effectively handling data not missing at random. However, its reliance on accurate probability estimation and instability of weights, especially in longitudinal studies, limits its robustness [40-42]. In contrast, imputation techniques estimate missing values based on patterns in the data, offering computational efficiency and suitability for clinical datasets with random missingness. Considering these factors, we adopted the k-Nearest Neighbors (KNN) imputation strategy. Initially, we excluded features with more than 30% missing values from the total dataset of 1080 rows (360 subjects × 3 visits) to ensure sufficient data for effective imputation

2. **Handle missing data based on imputation techniques**

Among the imputation techniques, we have considered two commonly used methods: k-Nearest Neighbors (KNN) and Multiple Imputation by Chained Equations (MICE).

- KNN imputation estimates missing values by identifying the most similar observations (neighbors) in the feature space and using their values to predict the missing ones. This method is computationally efficient and works well when the missing data is random and the relationships between features are well-captured by the proximity of observations.
- MICE performs multiple imputations by modeling each feature with missing values as a function of other features. It generates several different imputed datasets, providing more accurate estimates and accounting for the uncertainty in the imputed values. MICE is particularly useful when dealing with complex patterns of missing data, as it incorporates relationships between features in a more robust manner.

The results of important features and performance of LGMM model based KNN and MICE to handle missing data shown in **Table 1**. Based on the results in Table 1, the performance of KNN and MICE for handling missing data is comparable, making either method a viable choice for data processing.

Table 1: Comparison of the performance of the KNN and MICE methods in handling missing data

| Method | Results |
| --- | --- |
| KNN |   Figure: Feature importance scores based on tree-based method.  Performance of LGMM model:  Acc= 95.56% ± 1.72  Pre=80.21% ± 6.58  Spe= 97.39% ± 1.03  NPV=97.60% ± 2.12  Recall=82.64% ± 13.75 |
| MICE |   Figure: Feature importance scores based on tree-based method.  Performance of LGMM model:  Acc=95.65% ± 1.69  Pre=81.01% ± 7.88  Spe=97.50% ± 1.18  NPV = 97.60% ± 2.08  Recall=82.73% ± 13.88 |

3. **Sensitivity analyses**

Regarding the sensitivity analyses, we have included additional results showing the top 20 important features based on the tree-based approach with 5-fold cross-validation of the KNN imputation method for handling missing data, with parameters K=1, K=3, and K=5, as shown in Table 2. For K=3 and K=5, the error bars of the importance scores vary widely, while for K=1, the results are relatively stable. Therefore, K=1 is considered the optimal parameter.

We have also included a summary the results of sensitivity analyses in **Section III. Results** to find optimal K in this paragraph as follows:

Prior to feature engineering, we conducted simulations to identify the optimal K for the KNN imputation method to handle missing data. Using a tree-based approach with 5-fold cross-validation to calculate feature importance scores, we evaluated the performance of the KNN imputation method with K=1, K=3, and K=5. The results indicate that for K=3 and K=5, the error bars of the importance scores vary significantly, indicating instability in the imputation. In contrast, K=1 resulted in more stable and consistent results, suggesting that it is less prone to overfitting. Therefore, K=1 was selected as the optimal parameter, as it provides reliable imputation while maintaining the integrity of the dataset.

Table 2: The feature importance results calculated for three different KNN cases for handling missing data.

|  | **Features importance** |
| --- | --- |
| K=1 |  |
| K=3 |  |
| K=5 |  |

***Reviewer’s comment:***

**#3B - Clustering Validation: The robustness of GMM clustering is not validated using external metrics such as silhouette scores or Davies-Bouldin index. Adding these metrics would strengthen the credibility of the identified clusters.**

***Response****:*

Thank you for your comment. We have calculated and added Davies-Bouldin index and Silhouette scores in the **subsection III.C** as follows:

The Davies-Bouldin Index and the average Silhouette Score were calculated to validate the derived clustering [56]. The Davies-Bouldin Index assesses cluster compactness and separation by comparing the similarity between the most similar clusters, where a value close to 0 indicates well-separated and cohesive clusters [57]. Similarly, the Silhouette Score, ranging from -1 to 1, evaluates how well an object fits within its cluster compared to others. The average Silhouette Score of 0.867 and Davies-Bouldin Index of 0.567 of our clusters reflect strong cohesion within clusters and clear separation between them, confirming the effectiveness and reliability of the clustering approach. These results confirm that the identified clusters are both meaningful and reliable.

***Reviewer’s comment:***

**#3C - Generalizability: The cohort is largely middle-aged and homogenous, limiting the
applicability of findings to broader populations. Discuss the need for external validation
in diverse ethnic, socioeconomic, and geographic groups.**

***Response****:*

We appreciate the reviewer for pointing out our limitations regarding generalizability. Our study's reliance on the Wisconsin Sleep Cohort, while providing robust longitudinal data, does present limitations in population diversity. The cohort's middle-aged, demographically homogeneous composition may not fully capture CVD-OSA manifestations across different age groups, ethnic backgrounds, and healthcare systems. While our machine learning models achieved 80.5% diagnostic accuracy in predicting CVD outcomes, their performance needs validation in populations with different cardiovascular risk profiles, comorbidity patterns, and healthcare access. This validation is particularly important given the varying genetic predispositions to CVD and different baseline autonomic nervous system characteristics across populations. We have modified the discussion section to address these limitations by adding a comprehensive paragraph that details the specific validation needs for different age groups (<40 years, >75 years), clinical indicators (total cholesterol, LDL, diabetes), and transition patterns between Clusters 1 and 2, while also outlining future multi-center studies across diverse populations.

To address this, we have included the following in blue to **Section IV: Discussion**:

While our findings demonstrate strong predictive capability in the Wisconsin Sleep Cohort, we also acknowledge important limitations in generalizability. Our study population, predominantly middle-aged and demographically homogeneous, may not fully represent the spectrum of CVD-OSA manifestations across diverse populations. Although our models achieved 80.5% diagnostic accuracy in predicting CVD outcomes, several key aspects require further validation. First, the model's performance needs to be evaluated in younger patients (<40 years) who may present with different early markers of CVD progression, and in elderly populations (>75 years) who often have multiple competing comorbidities. Second, our clinical indicators analysis, which identified total cholesterol, LDL, and diabetes as key predictors, requires validation in populations with different genetic predispositions, varying baseline metabolic profiles, and diverse dietary and lifestyle patterns that may influence both CVD and OSA progression. Additionally, the transition patterns observed between Cluster 1 (slower CVD progression) and Cluster 2 (rapid onset/deterioration) need to be verified across populations with different socioeconomic barriers to healthcare access and in healthcare systems with varying approaches to CVD and OSA management. Future multi-center studies should evaluate these phenotypic clusters in populations with different baseline autonomic nervous system characteristics and environmental exposures that may affect sleep patterns, ensuring the broader clinical applicability of our phenotypic modeling approach. Moreover, integrating novel biomarkers and more detailed genetic information could improve the predictive power of the models and offer deeper insights into the biological underpinnings of the disease processes.

***Reviewer’s comment:***

**#3D - SMOTE (Synthetic Minority Over-sampling Technique), while helpful in addressing class imbalance, may introduce bias. Discuss its potential influence on model outcomes and provide validation to ensure generalizability.**

***Response****:*

We appreciate the reviewer's comment regarding the potential bias introduced by SMOTE and its influence on model outcomes, and we agree with your suggestions. In our research, SMOTE was used to address class imbalance by up-sampling the minority class by 100% during feature selection with 5-fold cross-validation (CV). For model evaluation, we used longitudinal data from WSCS, processed without SMOTE. To validate the robustness and generalizability of the model outcomes, 5-fold CV was employed, ensuring evaluation across multiple subsets of the dataset, which helps reduce the risk of overfitting to synthetic samples. Performance metrics, including Accuracy, Precision, Recall, Specificity, and NPV, were calculated across all 5 folds, consistently demonstrating the model's effectiveness. The results show high Accuracy (95.56%), Specificity (97.39%), Recall (82.64%), and Precision (80.21%), suggesting that SMOTE-based feature selection was effective in identifying potentially significant features. We have added the following to **Discussion section**:

Although SMOTE helps in addressing the class imbalance inherent in many clinical datasets, the technique generates synthetic data points that may not perfectly represent real-world conditions. Consequently, there is a risk that models trained on this data could be biased towards the over-represented class. To mitigate this risk, our analysis was complemented by stringent cross-validation during model evaluation. The data used for this phase did not include SMOTE-processed samples, ensuring that our evaluation metrics are based on actual clinical outcomes rather than synthetic approximations. Nonetheless, the initial use of SMOTE for feature selection could still influence the final model by potentially enhancing certain patterns that are not as pronounced in real-world data. It is crucial for future studies to consider these factors when using balancing techniques like SMOTE, especially in predictive modeling where the ultimate goal is to apply these findings in clinical settings.

***Reviewer’s comment:***

**#3E - Expand on the mechanistic pathways connecting OSA and CVD, particularly the role of intermittent hypoxia, oxidative stress, and autonomic dysfunction.**

***Response****:*

To expand on the mechanistic pathways connecting OSA and CVD, the amendments have been made to 2^nd^ paragraph of the **Introduction section**:

The mechanisms proposed to underlie this association include metabolic dysregulation [7, 14, 15], oxidative stress from intermittent hypoxia [2, 19, 20], and altered autonomic nervous system activity [2, 15, 20], which collectively contribute to cardiovascular pathology in OSA patients. The intermittent hypoxia and reoxygenation in OSA activate systemic inflammatory pathways, leading to the release of proinflammatory cytokines which exacerbate endothelial dysfunction and vascular remodeling, creating a pro-thrombotic environment that heightens the risk of CVD [7, 20]. Metabolic dysregulation in OSA patients often manifests as disrupted glucose and lipid profiles, particularly in individuals with coexisting obesity or diabetes. Meanwhile, oxidative stress from intermittent hypoxia accelerates the progression of atherosclerosis by promoting lipid peroxidation, smooth vascular muscle proliferation, and plaque instability [21, 22]. Sympathetic nervous system overactivity stimulated by the repetitive hypoxic episodes and arousals, coupled with reduced parasympathetic tone, also contributes to the cardiovascular burden by sustaining elevated heart rate and blood pressure, thereby imposing greater cardiac workload and risk of myocardial injury [2, 20, 23, 24]. However, the complexity of these interactions, compounded by a scarcity of longitudinal data, makes it challenging to untangle the temporal and causal relationships between these comorbid conditions [10, 13, 25]. This complexity is further magnified by the variability in individual patient responses and the multitude of confounding factors that obscure the direct impacts of these diseases on each other [12, 26].

***Reviewer’s comment:***

**#3F - Provide more explicit discussion on how phenotypes influence treatment strategies (e.g., CPAP therapy or lipid-lowering medications). This would bridge the findings to practical applications.**

***Response****:*

Thank you for your valuable suggestion regarding the need for more explicit discussion of treatment implications. We have addressed this by adding a comprehensive paragraph in the **Discussion section** that directly links our phenotypic findings to clinical applications, supported by key literature.

We have integrated findings from the SAVE trial [4] regarding CPAP therapy and recent European guidelines on combination lipid-lowering therapy [5] to provide evidence-based treatment recommendations. The new paragraph details:

1. A phenotype-specific treatment approach, differentiating between Cluster 1 patients who may benefit from conservative management and Cluster 2 patients who require more aggressive intervention.
2. How temporal progression patterns can inform the timing and intensity of interventions, enabling proactive treatment adjustments based on phenotypic classification.

These additions provide clinicians with practical, evidence-based guidance for implementing phenotype-specific treatment strategies.

Our clustering analysis identifies two distinct patient groups with direct implications for clinical decision-making:

- **Cardiovascular Stability Cluster (Cluster 1):** This cluster includes patients with more favorable health profiles, characterized by lower LDL cholesterol, fasting glucose, and fewer episodes of nocturnal hypoxia, indicating stable disease progression and effective management of CVD and OSA. These findings suggest the importance of maintaining current treatment regimens and focusing on preventive care, such as lifestyle modifications and routine monitoring. Patients in Cluster 1, characterized by slower CVD progression, may benefit from more conservative treatment approaches, with careful monitoring of CPAP adherence and standard cardiovascular risk management [81].
- **Cardiovascular Risk Cluster (Cluster 2):** This cluster includes patients with higher values of critical CVD biomarkers and more severe OSA symptoms, reflecting rapid disease progression and a need for intensive management strategies. For these patients, more aggressive lipid-lowering therapies, stricter glucose control, and potentially earlier initiation of lipid-lowering medications [82] as well as targeted interventions for sleep apnea, such as CPAP therapy, may be necessary.

These clustering insights support precision medicine by stratifying patients based on risk, enhancing decision-support tools to flag high-risk phenotypes, suggest personalized interventions, and prioritize resource allocation. For instance, patients identified in high-risk clusters could automatically trigger alerts or recommendations for further diagnostic tests or follow-up care.

**References:**

[4] R. D. McEvoy *et al.*, "CPAP for prevention of cardiovascular events in obstructive sleep apnea," *New England Journal of Medicine*, vol. 375, no. 10, pp. 919–931, 2016.

[5] K. K. Ray *et al.*, "Combination lipid-lowering therapy as first-line strategy in very high-risk patients," *European Heart Journal*, vol. 43, no. 8, pp. 830–833, 2022.

***Reviewer’s comment:***

**#3G - Propose actionable recommendations for clinical practice, such as thresholds for monitoring nocturnal hypoxia or specific lipid levels based on phenotypic risk stratification.**

***Response****:*

We thank the reviewer for their valuable suggestion. To enhance the clinical utility of our work, we have proposed the following actionable recommendations and included them in **Section IV: Discussion**:

Monitoring nocturnal hypoxia, defined as oxygen saturation below 90% of sleep time, can also aid in identifying high-risk individuals, as prolonged nocturnal desaturation is associated with increased risks of CVD [83]. OSA screening is recommended for patients with poorly controlled hypertension, heart failure, recurrent atrial fibrillation, or a history of stroke, with CPAP therapy prioritized for severe OSA cases. Integrating risk stratification and treatment strategies into routine cardiovascular practice not only supports personalized care but also addresses the under-recognition of OSA and its critical role in CVD outcomes.

Furthermore, the temporal progression patterns identified in our study could inform the timing and intensity of interventions, allowing clinicians to proactively adjust treatment plans based on a patient's phenotypic classification. Building on these insights, the analysis of Tables A5 and A6 provides the following actionable recommendations for patients in Group 7 (long-term CVD):

- **Cluster 1 recommendations**: Patients in Cluster 1 should focus on maintaining their current metrics within a healthy range, including lipid levels, AHI, and NREM-AHI. Regular check-ups and proactive management of these indicators are important to prevent a potential transition to Cluster 2.
- **Cluster 2 recommendations:** Patients in Cluster 2 should undergo regular monitoring and targeted medical interventions to control lipid levels and improve respiratory health. To facilitate the transition to Cluster 1, patients should focus on comprehensive lifestyle changes, including adopting a heart-healthy diet, increasing physical activity, and managing stress, as well as adopting specific treatments as needed for cholesterol management, weight loss, or respiratory metrics. We recommend that patients aim for the following target values: total cholesterol between 123.50 mg/dL and 155.33 mg/dL, with a target of 139.42 mg/dL; LDL between 56.25 mg/dL and 81 mg/dL, with a target of 68.63 mg/dL; triglycerides between 102.00 mg/dL and 108.33 mg/dL, with a target of 105.17 mg/dL; and AHI between 12.93 and 22.60, with a target of 17.77.

Recent research indicates that extremely low or high LDL-C levels might be linked to higher mortality rates. LDL levels below 50 mg/dL or above 130 mg/dL have been linked to a higher risk of mortality in patients with coronary artery disease [84], highlighting the relationship between extreme LDL levels and all-cause death rates. Additionally, severe OSA (AHI ≥30) has been associated with a higher risk of both all-cause and cardiovascular mortality [3]. However, it is important to note that the baseline characteristics are not fully aligned across the sub-groups. This heterogeneity limits our ability to define specific thresholds for clusters or to provide definitive recommendations regarding remaining in or transitioning to a preferable healthy cluster. Consequently, while we can offer general suggestions on managing key indicators such as lipid levels and respiratory health based on our findings, further studies with more consistent baseline data are needed to establish clearer thresholds and provide precise recommendations for achieving optimal cluster outcomes.

***Reviewer’s comment:***

**#3H - Highlight potential integration into clinical workflows, such as embedding the model into electronic health records or decision-support tools to guide patient management.**

***Response****:*

Thank you for the reviewers' valuable suggestion regarding the integration of our model into clinical workflows. We have included perspectives in the revised manuscript in **Section IV: Discussion**, as follows:

Our proposed research, which combines the LGMM for patient trajectory analysis and clustering using t-SNE and GMM, holds significant potential for integration into clinical workflows. Specifically, the Logistic Mixed-Effects Model could be embedded into electronic health records (EHR) utilizing 20 selected features to provide predictions of CVD outcomes, enabling clinicians to dynamically track patient trajectories and make timely interventions. t-SNE and GMM-based clustering results could further classify patients into phenotypic groups directly within EHR systems, offering a visual and data-driven understanding of patient profiles.

These clustering insights support precision medicine by stratifying patients based on risk, enhancing decision-support tools to flag high-risk phenotypes, suggest personalized interventions, and prioritize resource allocation. For instance, patients identified in high-risk clusters could automatically trigger alerts or recommendations for further diagnostic tests or follow-up care.

**Minor Comments:**

***Reviewer’s comment:***

**#3I - Introduction: The introduction is comprehensive but could further highlight gaps in existing predictive models and emphasize how this study addresses those gaps.**

***Response****:*

Thank you for the reviewer's comment. We have included additional explanations on the current gaps and how we address them in our study as follows (additional edits were made to the previous manuscript to improve articulation). Specifically, the additions place further emphasis on the lack of studies focusing on the progression of OSA-CVD comorbidities, elaborated on the current methods used and the shortcomings of these methods, and pinpointed how our model overcomes the inadequate handling of multifactorial influences on OSA-CVD development.

Longitudinal research probing the evolution and interaction of OSA and CVD remains scarce.

For instance, while odds ratio or linear regression models effectively identify associations and predictive markers, they are inherently static and cannot explicitly handle the dynamic changes critical to understanding disease progression [27, 29, 33]. Similarly, more conservative approaches like survival analysis models capture certain longitudinal aspects of the OSA-CVD relationship, but fail to account for repeated measurements or explore phenotypes in depth [34-37]. This leaves significant gaps in comprehensively modeling disease trajectories and the heterogeneity of patient experiences. By leveraging advanced feature engineering, our study also incorporates the multifactorial influences driving OSA-CVD interactions, ensuring a more comprehensive examination of drivers behind disease progression.

***Reviewer’s comment:***

**#3J - Mechanistic Insights: While the study identifies important predictors, it lacks a deeper exploration of their biological roles. Including a brief discussion on the interplay between OSA severity, vascular changes, and metabolic dysregulation would enhance the narrative.**

***Response****:*

Thank you for the valuable insights. We have addressed this by adding a paragraph to the **discussion section** that explores the biological mechanisms linking OSA severity, vascular changes, and metabolic dysregulation. The new paragraph explains how recurrent intermittent hypoxia triggers oxidative stress and systemic inflammation leading to endothelial dysfunction, while sleep fragmentation disrupts glucose homeostasis through insulin resistance. We particularly connect these mechanistic pathways to our phenotypic clustering findings, explaining how the synergistic effects of vascular inflammation, sympathetic overactivity, and metabolic dysfunction may drive the rapid disease progression observed in Cluster 2 patients. This addition strengthens the biological foundation of our phenotypic modeling approach while maintaining focus on the study's primary objectives.

Mechanistically, the interplay between OSA severity, vascular dysfunction, and metabolic dysregulation provides insight into the observed CVD progression patterns. Recurrent intermittent hypoxia induces oxidative stress and systemic inflammation, leading to endothelial dysfunction and arterial stiffness [79], while sleep fragmentation disrupts glucose homeostasis through increased insulin resistance [80]. These synergistic pathways - vascular inflammation, sympathetic overactivity, and metabolic dysfunction - may explain the aggressive disease trajectory in Cluster 2. In particular, patients in Cluster 2 likely experienced heightened sympathetic activation and severe nocturnal hypoxemia, intensifying the inflammatory cascade and metabolic disruption, which resulted in their observed rapid onset and deterioration of cardiovascular conditions compared to Cluster 1.

***Reviewer’s comment:***

**#3K - Limitations Section: The limitations section should discuss potential confounders, such as lifestyle factors (e.g., smoking, alcohol use, and caffeine intake), which were not integrated into the analysis. Additionally, self-reported data for certain variables should be acknowledged as a limitation.**

***Response****:*

Thank you for your valuable suggestion. While we mentioned potential confounders, we did not delve into the impact of them in our **discussion** and thus have included further elaboration on this along with possible techniques or actions that could be taken in future work. We have also included in the discussion our acknowledgement of self-reported data as a limitation.

The absence of key lifestyle factors, such as smoking, alcohol use, and caffeine intake, due to the feature selection process may result in unaccounted variability in the data, potentially obscuring the true relationships between the observed features and cardiovascular risk. Given the nature of the current dataset, it was not possible to address these confounders comprehensively. However, their potential impact on the clustering process, such as altering the composition and interpretation of the identified phenotypic clusters. Similarly, their absence may limit the ability of the model to fully capture the multifactorial etiology of cardiovascular conditions. Future research should focus on integrating more comprehensive datasets that include these lifestyle factors and employing causal inference methods to address confounding more robustly. Techniques such as structural equation modeling or propensity score adjustment could help isolate the effects of these unmeasured variables, providing a more nuanced understanding of the phenotypic clusters and their relationship with cardiovascular outcomes.

Our current work relies on self-reported data, particularly for variables related to medical history, which are subjected to recall bias and may impact the accuracy of the findings.

**APPENDIX**

Table A3: Comparative analysis of variables for healthy patients from visit 2 who tend to move to Cluster 1 or Cluster 2 in visit 3 within Group 1

|  | **G1V2HV3C1** | | **G1V2HV3C2** | | | |
| --- | --- | --- | --- | --- | --- | --- |
|  | **Visit2** | **Visit 3**  **(Cluster1)** | **Visit2** | | **Visit 3**  **(Cluster2)** | |
|  | **Subjects = 4** | | **Subjects =9** | | | |
| Cholesterol medication | No, N=4 100% | Yes, N=4  100% | No, N=5 55.56% | Yes,N=4  44.44% | No, N=2  22.22% | Yes, N=7  77.78% |
| MACE1 | 0 | 4(100) | 0 | 0 | 1(50) | 5(71.43) |
| MACE1 treatment | 0 | 3(75) | 0 | 0 | 1(50) | 1(14.28) |
| MACE3 | 0 | 0 | 0 | 0 | 1(50) | 2(28.57) |
| MACE3 treatment | 0 | 0 | 0 | 0 | 0 | 0 |
| apnea | 0 | 0 | 0 | 1(25) | 1(50) | 3(42.86) |
| apnea treatment | 0 | 0 | 0 | 1(25) | 1(50) | 3(42.86) |
| total cholesterol | 236.75  (20.42) | 130.50  (14.20) | 224.00  (53.16) | 189.00  (21.37) | 221.00  (43.84) | 169.00  (22.11) |
| ldl | 160.25  (31.33) | 71.75  (13.62) | 130.20  (58.83) | 104.50  (16.74) | 107.50  (2.12) | 89.29  (13.49) |
| triglycerides | 111.75  (62.71) | 61.75  (22.34) | 258.60  (115.33) | 138.75  (4.50) | 358.00  (247.49) | 172.43  (41.18) |
| nremahi | 3.95  (4.47) | 4.83  (4.01) | 11.36  (10.28) | 11.18  (7.96) | 19.05  (17.04) | 11.19  (10.34) |
| ahi | 4.05  (4.10) | 5.28  (2.53) | 16.00  (13.10) | 13.73  (4.05) | 26.15  (21.71) | 12.59  (9.23) |
| hipgirthm | 100.58  (6.13) | 97.58  (6.36) | 121.96  (14.69) | 121.95  (20.68) | 135.50  (21.92) | 116.37  (12.90) |
| diabetes_med | 0 | 0 | 2(40) | 1(25) | 1(50) | 3(42.86) |
| arthritis_ynd | 0 | 0 | 4(80) | 1(25) | 2(100) | 5(71.43) |
| bmi | 27.73  (3.16) | 26.48  (2.26) | 36.48  (6.06) | 39.38  (11.44) | 42.00  (8.63) | 34.84  (7.37) |
| age | 57.50  (4.80) | 61.50  (4.43) | 59.80  (12.32) | 57.75  (6.34) | 53.50  (0.71) | 66.29  (9.27) |
| creatine | 1.15  (0.19) | 1.01  (0.22) | 0.98  (0.30) | 1.03  (0.17) | 0.86  (0.20) | 1.06  (0.32) |
| waitsthip | 0.91  (0.09) | 0.95  (0.08) | 0.92  (0.09) | 0.96  (0.12) | 0.92  (0.06) | 0.98  (0.07) |
| Zung index | 37.81  (5.44) | 38.13  (4.27) | 43.25  (9.30) | 41.88  (7.81) | 56.88(7.95) | 41.96(5.04) |

Table A4: Comparative analysis of variables for healthy patients from visit 1 who tend to move to Cluster 1 or Cluster 2 in visit 2 within Group 3

|  | **G3V1HV2C1** | | **G3V1HV2C2** | | | |
| --- | --- | --- | --- | --- | --- | --- |
|  | **Visit1** | **Visit 2**  **(Cluster 1)** | **Visit1** | | **Visit 2**  **(Cluster 2)** | |
|  | **Subjects = 4** | | **Subjects = 5** | | | |
| Cholesterol  medication | No, N=4  (100%) | Yes, N=4  (100%) | No, N=4  (80%) | Yes, N=1  (20%) | No, N=1  (20%) | Yes, N=4  (80%) |
| MACE1 | 0 | 3 (75) | 0 | 0 | 1 | 3(75) |
| MACE1 treatment | 0 | 4(100) | 0 | 0 | 0 | 4(100) |
| MACE3 | 1(25) | 0 | 2 | 0 | 1 | 1(25) |
| MACE3 treatment | 0 | 0 | 0 | 0 | 0 | 1(25) |
| apnea | 1(25) | 1(25) | 1(25) | 1 | 1 | 2(50) |
| apnea treatment | 1(25) | 1(25) | 1(25) | 1 | 1 | 2(50) |
| total cholesterol | 209.33  (21.73) | 133.00  (11.17) | 203.25  (47.68) | 216.00 | 169.00 | 162.00  (19.85) |
| ldl | 130.00(28.35) | 59.25(7.27) | 116.50(49.20) | 144.00 | 105.00 | 88.25(29.43) |
| triglycerides | 176.00  (95.09) | 89.00  (17.26) | 230.25  (143.22) | 154.00 | 148.00 | 201.00  (137.64) |
| nremahi | 21.53  (25.49) | 22.58  (21.86) | 36.28(49.14) | 2.20 | 5.20 | 29.60(40.38) |
| ahi | 23.67  (24.85) | 24.60  (22.40) | 39.73(48.74) | 2.80 | 10.70 | 31.55(38.67) |
| hipgirthm | 98.43  (9.40) | 99.83(6.34) | 116.63(11.16) | 102.30 | 133.00 | 110.45  (12.04) |
| diabetes_med | 0 | 1(25) | 0 | 1 | 0 | 1(25) |
| arthritis_ynd | 1(25) | 1(25) | 2(50) | 0 | 1 | 2(50) |
| bmi | 26.93(3.00) | 26.40(4.34) | 35.63(6.01) | 30.60 | 41.20 | 34.28(6.96) |
| age | 60.67(8.62) | 66.00(7.87) | 59.50(9.18) | 65.00 | 72.00 | 62.25(7.04) |
| creatine | 1.27(0.06) | 1.10(0.08) | 1.03(0.21) | 1.10 | 1.40 | 0.96(0.16) |
| waitsthip | 1.00(0.06) | 0.98(0.10) | 0.95(0.09) | 1.02 | 0.89 | 0.97(0.12) |
| Zung index | 42.50(6.50) | 42.50(8.10) | 46.25(18.74) | 45.00 | 33.75 | 41.25(10.46) |

Table A5: Comparative analysis of variables for patients from visit 1 who tend to move to Cluster 1 or Cluster 2 in visit 2 within Group 7.2 and Group 7.3

|  | **G7V1C2V2C1** | | | **G7V1C2V2C2** | |
| --- | --- | --- | --- | --- | --- |
|  | **Visit1**  **(Cluster 2)** | | **Visit 2**  **(Cluster 1)** | **Visit1**  **(Cluster 2)** | **Visit 2**  **(Cluster 2)** |
|  | **Subjects = 7** | | | **Subjects =6** | |
| Cholesterol  medication | No, N=1  14.29% | Yes, N=6  85.71% | Yes, N=7  100% | Yes, N=6  100% | Yes,N=6  100% |
| MACE1 | 1 | 6(100) | 7(100) | 5(83.33) | 5(83.33) |
| MACE1 treatment | 1 | 4(66.67) | 7(100) | 5(83.33) | 5(83.33) |
| MACE3 | 0 | 2(33.33) | 2(28.57) | 2(33.33) | 3(50) |
| MACE3 treatment | 0 | 0 | 0 | 0 | 0 |
| apnea | 1 | 0 | 2(28.57) | 0 | 1(16.67) |
| apnea  treatment | 1 | 0 | 2(28.57) | 0 | 1(16.67) |
| total cholesterol | 157.00 | 170.33(30.69) | 146.43(22.86) | 173.67(24.38) | 162.50(20.90) |
| ldl | 76.00 | 84.67(18.92) | 72.00(23.01) | 86.83(25.24) | 82.00(18.92) |
| triglycerides | 239.00 | 179.67(33.69) | 106.14(25.16) | 232.17(51.13) | 189.00(52.49) |
| nremahi | 22.10 | 14.80(21.57) | 14.13(10.19) | 21.18(30.19) | 14.55(18.90) |
| ahi | 23.20 | 18.48(21.74) | 17.07(11.35) | 23.18(32.00) | 17.30(21.76) |
| hipgirthm | 112.80 | 105.93(9.99) | 104.54(6.08) | 115.05(5.79) | 111.13(10.88) |
| diabetes_med | 0 | 1(16.67) | 2(28.57) | 3(50) | 4(66.67) |
| arthritis_ynd | 1 | 2(33.33) | 4(57.14) | 3(50) | 2(33.33) |
| bmi | 33.40 | 29.63(4.39) | 30.00(3.73) | 36.42(5.77) | 35.32(7.30) |
| age | 70.00 | 59.50(7.31) | 65.14(7.67) | 61.00(6.54) | 65.00(6.57) |
| creatine | 1.30 | 1.05(0.20) | 1.04(0.19) | 0.90(0.15) | 0.98(0.18) |
| waitsthip | 1.01 | 0.97(0.07) | 0.99(0.07) | 1.00(0.12) | 1.01(0.11) |
| Zung index | 38.75 | 43.54(6.63) | 40.54(8.00) | 43.54(8.85) | 42.92(5.16) |

Table A6: Comparative analysis of variables for patients from visit 2 who tend to move to Cluster 1 or Cluster 2 in visit 3 within Group 7.2 and Group 7.3

|  | **G7V2C1V3C1** | | **G7V2C1V3C2** | |
| --- | --- | --- | --- | --- |
|  | **Visit 2**  **(Cluster 1)** | **Visit 3**  **(Cluster 1)** | **Visit 2**  **(Cluster 1)** | **Visit 3 (Cluster2)** |
|  | Subjects = 4 | | Subjects =3 | |
| Cholesterol medication | Yes, N=4  100% | Yes, N=4  100% | Yes, N=3  100% | Yes, N=3  100% |
| MACE1 | 4(100) | 4(100) | 3(100) | 3(100) |
| MACE1 treatment | 4(100) | 4(100) | 3(100) | 3(100) |
| MACE3 | 2(50) | 2(50) | 0 | 0 |
| MACE3 treatment | 0 | 0 | 0 | 0 |
| apnea | 2(50) | 3(75) | 0 | 0 |
| apnea treatment | 2(50) | 2(50) | 0 | 0 |
| total cholesterol | 139.75(20.09) | 123.50(14.55) | 155.33(27.47) | 158.33(15.89) |
| ldl | 65.25(9.22) | 56.25(9.32) | 81.00(35.34) | 88.00(16.37) |
| triglycerides | 104.50(35.29) | 102.00(13.98) | 108.33(4.16) | 171.67(68.54) |
| nremahi | 11.45(9.64) | 18.80(29.21) | 17.70(11.77) | 19.53(21.73) |
| ahi | 12.93(8.56) | 20.43(29.06) | 22.60(14.00) | 25.03(22.19) |
| hipgirthm | 107.20(1.80) | 107.20(1.59) | 101.00(8.54) | 101.17(10.25) |
| diabetes_med | 2(50) | 2(50) | 0 | 0 |
| arthritis_ynd | 2(50) | 3(75) | 2(66.67) | 2(66.67) |
| bmi | 30.80(2.18) | 31.13(1.67) | 28.93(5.62) | 29.97(6.43) |
| age | 67.75(5.68) | 71.50(5.32) | 61.67(9.81) | 66.67(9.81) |
| creatine | 1.08(0.25) | 1.11(0.28) | 1.00(0.10) | 1.03(0.33) |
| waitsthip | 1.00(0.04) | 1.05(0.06) | 0.96(0.11) | 1.02(0.09) |
| Zung index | 37.81(8.92) | 41.25(10.75) | 44.17(6.17) | 41.25(9.44) |
